# The Efficacy of Omega-3 Fatty Acids as the Monotherapy for Depression: A Randomized, Double-Blind, Placebo-Controlled Pilot Study

**DOI:** 10.3390/nu16213688

**Published:** 2024-10-29

**Authors:** Suet-Kei Wu, Kai-Jie Yang, Wen-Chun Liu, Ikbal Andrian Malau, Halliru Zailani, Cheng-Ho Chang, Shih-Yi Huang, Jane Pei-Chen Chang, Wei-Che Chiu, Kuan-Pin Su

**Affiliations:** 1 Mind-Body Interface Research Center (MBI-Lab), China Medical University Hospital, Taichung 404, Taiwan; wskei23@gmail.com (S.-K.W.); kjyang0130@gmail.com (K.-J.Y.); ikbalgan@gmail.com (I.A.M.); halliruzyln55@gmail.com (H.Z.); peko80@gmail.com (J.P.-C.C.); 2 Graduate Institute of Nutrition, China Medical University, Taichung 404, Taiwan; 3 Department of Nursing, National Tainan Junior College of Nursing, Tainan 700, Taiwan; graceliu8911@gmail.com; 4 Graduate Institute of Biomedical Sciences, China Medical University, Taichung 404, Taiwan; 5 Department of Psychiatry, Kaohsiung Veterans General Hospital, Kaohsiung 813, Taiwan; kvjang58@gmail.com; 6 School of Nutrition and Health Sciences, Taipei Medical University, Taipei 110, Taiwan; sihuang@tmu.edu.tw; 7 College of Medicine, China Medical University, Taichung 404, Taiwan; 8 Child and Adolescent Psychiatry Division, Department of Psychiatry, China Medical University Hospital, Taichung 404, Taiwan; 9 Department of Psychiatry, Cathay General Hospital, Taipei 106, Taiwan; 10 School of Medicine, Fu Jen Catholic University, Taipei 242, Taiwan; 11 An-Nan Hospital, China Medical University, Tainan 709, Taiwan

**Keywords:** depression, monotherapy, *n*-3 PUFAs, RCT

## Abstract

Objective: Omega-3 polyunsaturated fatty acids (*n*-3 PUFAs) have demonstrated protective effects in major depressive disorder (MDD) patients receiving antidepressant treatment. However, there have been a few double-blind randomized controlled trials focused on *n*-3 PUFAs as monotherapy in MDD, and the outcomes have been mixed. This study aimed to assess the clinical effects of *n*-3 PUFAs monotherapy in patients with MDD. Methods: A total of 60 patients with MDD participated in this 12-week double-blind randomized controlled trial. They were randomized to either the *n*-3 PUFAs group (n = 30; 3.2 g of eicosapentaenoic acid, EPA and docosahexaenoic acid, DHA per day) or the placebo group (n = 30; 3.2 g of soybean oil per day). The severity of depression was evaluated using the Hamilton Rating Scale for Depression (HRSD). Results: The *n*-3 PUFAs group had a significantly lower HRSD score compared with the placebo group at week 4 (*p* = 0.004), week 6 (*p* = 0.006), week 8 (*p* = 0.004), and week 12 (*p* = 0.01). The *n*-3 PUFAs group showed slightly higher rates for both remission (26.7% vs. 10%, *p* = 0.095) and response (23.3% vs. 6.7%, *p* = 0.145) compared with the placebo group at week 12, but these differences did not reach statistical significance. Conclusions: These findings suggested that monotherapy of *n*-3 PUFAs could improve depression and potentially serve as an alternative option for MDD patients.

## 1. Introduction

Major depressive disorder (MDD) is a serious yet prevalent psychiatric disorder that has gradually become a chronic disease [1]. Overall, MDD affects 322 million people across the whole world [2]. The chronic nature of MDD and its related financial strain highlight the necessity for successful treatment options [3]. Currently, there are multiple types of treatment options but their efficacy is limited, with only one-third of MDD patients experiencing full remission with the first-line antidepressant agents [4,5]. Therefore, there is an unmet clinical need for safe and effective therapeutic approaches, such as electroconvulsive therapy (ECT) [6] and nutraceuticals [7]. Recently, omega-3 polyunsaturated fatty acids (*n*-3 PUFAs) have been reported with outstanding treatment outcomes in various disorders [8,9].

*n*-3 PUFAs, essential fatty acids that can only be obtained from food, are characterized by a double bond on the three carbon atoms at the end of the carbon chain [10]. Eicosapentaenoic acid (EPA) and docosahexaenoic acid (DHA) are major components of *n*-3 PUFAs and possess a variety of anti-inflammatory properties, [11,12] thereby resulting in the alleviation of inflammation across various disorders, including rheumatoid arthritis and MDD [13,14,15]. Clinical and epidemiologic studies have shown that inadequate *n*-3 PUFA levels may increase the chance of developing MDD [16,17,18]. Moreover, patients with depression often exhibit a lower level of omega-3 fatty acids than healthy individuals, particularly individuals with MDD who are unresponsive to standard antidepressants [19,20,21]. Therefore, researchers began to investigate the potential of *n*-3 PUFAs supplementation to augment other therapeutic approaches in alleviating depressive symptoms [19,22,23,24,25,26]. This builds on evidence from previous research suggesting the use of *n*-3 PUFAs as an oral supplement to improve depressive symptoms [9,27,28], and a recent cohort study linking *n*-3 PUFAs levels with protection against developing or experiencing persistent depressive episodes in adults [29]. This is further strengthened by studies on other mental disorders, such as ADHD [30], anxiety disorders [31], and bipolar disorder [32], which all presented improvement in the associated symptoms [33,34]. Moreover, the prescription of *n*-3 PUFAs (RxOME3FAs) has emerged as a therapeutic alternative in several countries, and they are generally safe and well tolerated [35].

Studies on the effect of *n*-3 PUFAs in MDD reported differing results. Specifically, recent clinical studies have presented the benefits of using *n*-3 PUFAs as monotherapy [36,37]. A double-blind, randomized and controlled, 8-week *n*-3 PUFAs monotherapy clinical trial reported that the *n*-3 PUFAs group presented an improvement in depressive symptoms among pregnant women [38]. On the contrary, some studies reported no superior therapeutic efficacy of *n*-3 PUFAs over placebo in MDD [39,40,41]. These inconsistent findings may be attributed to several factors, including variations in study duration, *n*-3 PUFAs dosages, and sample size. Furthermore, in terms of the study duration, adults with MDD were administered 1 g of *n*-3 PUFAs enriched with EPA and DHA as monotherapy, respectively, for 8 weeks, but no significant differences were observed across the groups [42]. Previous studies obtained inconsistent results on the dosage of *n*-3 PUFAs: some present the results that high-dose (>2 g/day) *n*-3 PUFAs supplementation may be more beneficial than low-dose supplementation with regard to MDD [43], and another meta-analysis stated that a low dose (<1 g/day) of *n*-3 PUFAs with EPA ≥ 60% resulted in an improvement in depression symptoms among MDD patients [44]. Therefore, further analysis of *n*-3 PUFAs dosage and treatment duration is required to reveal the relationship between *n*-3 PUFAs and depression at the clinical stage. Due to the shortcomings of the previous trials, this study aimed to evaluate the clinical impact of high-dose (3 g/day) *n*-3 PUFAs monotherapy on depression severity in patients with MDD through a 12-week, randomized, double-blind, placebo-controlled trial. 

## 2. Materials and Methods

### 2.1. Study Design

This study was a 12-week, parallel-group, double-blind randomized controlled trial to discover the monotherapy influence of *n*-3 PUFAs in depressed adults. The participants were recruited from the Outpatient Psychiatry Department of China Medical University Hospital and Cathay General Hospital, Taipei, Taiwan. Institutional review board approvals were obtained at both sites (IRB number: DMR93-IRB-87 and CT9336). This study was registered at the ClinicalTrials.gov (NCT00816322) and was conducted between 22 December 2004 to 21 December 2005, following the provision of the Declaration of Helsinki. Our study followed the CONSORT Guideline (See Supplementary File S1).

### 2.2. Study Participants

Adults aged 18 to 65 years with a confirmed diagnosis of MDD according to the fifth edition of The Diagnostic and Statistical Manual of Mental Disorders (DSM-IV) [45], and with 21-item version of Hamilton Rating Scale Depression (HRSD-21) score > 18 but no pharmacological and psychosocial intervention in the previous 8 weeks, were enrolled. Patients were excluded if they had a history of mental disorders other than major depressive disorder, such as schizophrenia, bipolar disorder, psychotic disorders, organic mental disorders, and substance use disorders, or if they had acute psychotic states or strong suicidal intentions. The eligible patients read and signed informed consent to participate in the study.

### 2.3. Randomization and Intervention

Sixty eligible patients were asked to read and sign informed consent. They were randomly assigned with a 1:1 ratio to the *n*-3 PUFAs group and placebo group using computer-generated randomization with block randomization. The randomization list was prepared by an investigator who is not engaged in clinical practice. During the whole trial, the participants would not receive any other treatments, such as antidepressants or psychosocial therapy. Participants in the *n*-3 PUFAs group were provided with fish oil capsules, each containing 420 mg EPA, 220 mg of DHA, 0.2 mg of tertiary-butylhydroquinone, and 2 mg of vitamin E. The *n*-3 PUFAs were extracted from the menhaden fish. Participants were asked to take five capsules of fish oil per day, which is a total of 3.2 g PUFAs (EPA, 2.1 g and DHA, 1.1 g) per day. The placebo group received soybean oil and was instructed to take 3.2 g per day, which amounted to five capsules per day. Both *n*-3 PUFAs and placebo capsules were processed similarly, including vacuum deodorization and orange flavoring, resulting in identical appearance, smell and taste. Moreover, both the patients and the researchers (responsible for clinical assessment) were blinded to the allocation. The criteria for study termination were defined as follows: a high risk of suicide (HRSD-21 Suicide Item Score ≥ 3); hospitalization necessity, evaluated by the physicians; patients’ determination to end the treatment; and the appearance of side effects with a structured symptom-checking list.

### 2.4. Clinical Assessment

Patients’ demographic data were collected at their first visit. The patients’ depression symptomatologies were evaluated using the HRSD, a 21-item, structured questionnaire, through an interview by well-trained clinical professionals, and simultaneously examined by the supervising psychiatrists [46]. The HRSD assessment was conducted at weeks 0, 2, 4, 6, 8, and 12 for each participant. The clinician was blinded during the clinical assessment.

### 2.5. Biochemical Analysis

Fasting venous blood samples were collected from the patients at baseline (week 0) and week 12 of the study to determine their erythrocytes *n*-3 PUFAs composition, blood lipid profile (including cholesterol, triglycerides, low-density lipoprotein (LDL) cholesterol and high-density lipoprotein (HDL) cholesterol) and other biochemical parameters related to liver and kidney functions. We evaluated the level of these biochemical parameters: aspartate aminotransferase (AST/GOT), alanine aminotransferase (ALT/GPT), blood urea nitrogen (BUN), creatinine, albumin, prothrombin time (PT), activated partial thromboplastin time (APTT), glucose, and prolactin. During the laboratory testing, the participants’ information was kept confidential, and the collected samples were measured by the investigators, who were blinded to the participants’ information. Thin-layer chromatography and gas chromatography were techniques used for analyzing the erythrocytes *n*-3 PUFAs composition, by comparing the retention time to the standard fatty acid methyl esters. The detailed procedures were adapted from previously published studies [47,48].

### 2.6. Sample Size Estimation

The sample size estimation for each group (n) was estimated using the following standard equation that includes the difference between treatment and placebo groups ($\mu_{1}-\mu_{2}$) and population variance ($\sigma^{2}$). The formula used traditional multiplier (a+b) to represent alpha (0.05) and beta (0.08), respectively. A priori power calculations indicated that a sample size of 16 participants in each group would have 80% power (*p* < 0.05) to detect a decrease of 2 points in the HRSD when the population variance was assumed to be 3 [47], but we increased the sample size to 30 participants in each group to account for anticipated dropouts.

$$n=\frac{2[{\left(a+b\right)}^{2}\sigma^{2}]}{(\mu_{1}-\mu_{2}{)}^{2}}$$

### 2.7. Statistical and Outcomes Analysis

An intention-to-treat (ITT) analysis was used to evaluate all the pre-treatment and post-treatment effects outcomes. The multiple imputation method was used to estimate missing values in order to mitigate potential biases related to missing data. Participants with at least one post-treatment assessment were included in the analysis following an intention-to-treat approach. Participants with no post-treatment data were excluded from the analysis. For the primary outcomes, the efficacy of *n*-3 PUFAs as monotherapy on the alternation of HRSD scores from baseline to week 12 was examined using the mixed model, repeated measures analysis approach. This model included the treatment group, treatment week, and their interaction as a fixed effect, subject as a random effect, and baseline score as covariates. As for the secondary outcome measures, the response rate was defined as a reduction in HRSD score of at least 50% from baseline to week 12, and the remission rate was defined based on the participant having an HRSD score of less than 8. Comparisons of remission rate and response rate were analyzed using an χ^2^ (chi-squared) test or Fisher’s exact test. An independent *t*-test or a Mann–Whitney U test was carried out to examine the differences between groups for continuous variables, including age, HRSD scores, and other biochemical parameters. For the statistical measures of this study, the level of significance was set as *p* < 0.05. All the data were analyzed with SPSS statistical software version 22.

## 3. Results

### 3.1. Patient Selection

**Figure 1:**
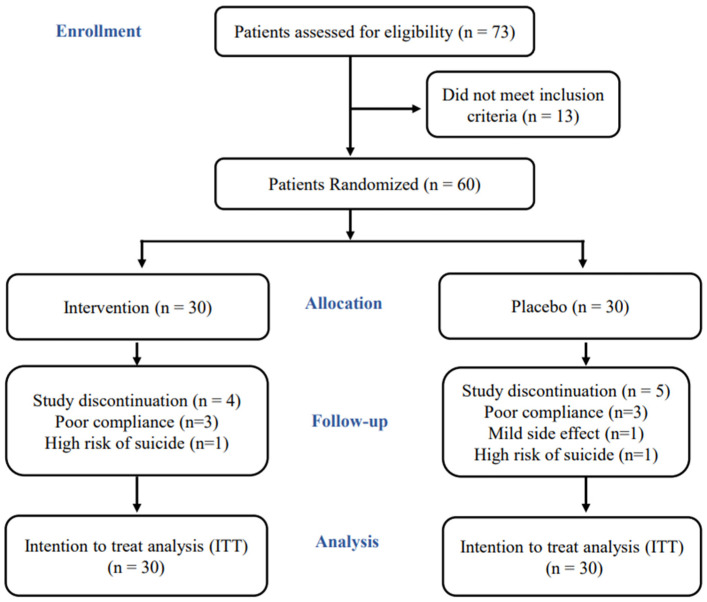
Consolidated standards of reporting trials (CONSORT) patient selection flow chart of the study. This flow chart contains the screening process, randomization, treatment, and follow-up of the patients.

The patient selection flowchart is shown in Figure 1. A total of seventy-three patients were assessed for eligibility, and only sixty of them were eligible to participate in the study. Among these patients, thirty patients were randomized to the *n*-3 PUFAs group while the other thirty were randomized to the placebo group and were followed up. In the *n*-3 PUFAs group, four patients discontinued the study: three due to poor compliance and one due to a high risk of suicide. On the contrary, five patients in the placebo group discontinued the study, of which three discontinued due to poor compliance, one due to mild side effects, and one due to a high risk of suicide.

### 3.2. Patient Characteristics

A total of sixty MDD patients were randomized to either the *n*-3 PUFAs group (n = 30) or the placebo group (n = 30). The basic demographic information, such as age, sex, and education level as well as outcomes, including HRSD scores, are presented in Table 1.

### 3.3. Comparison of the HRSD Score

**Figure 2:**
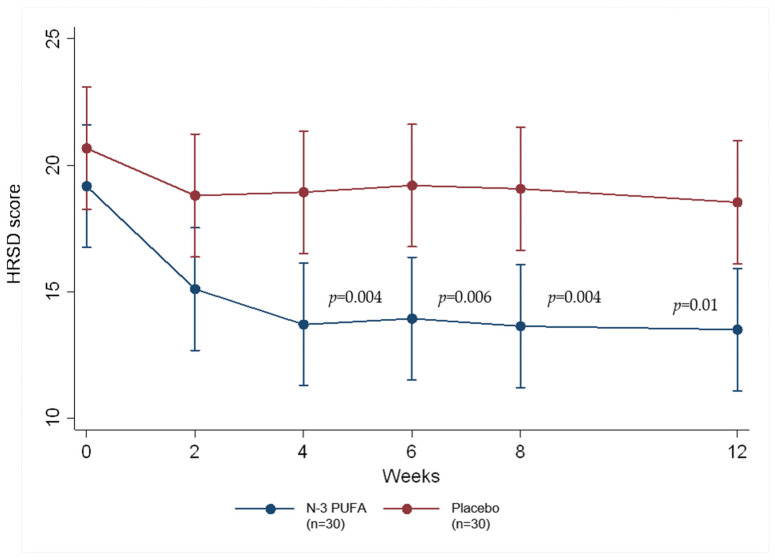
Evolution of the 21-item Hamilton Rating Scale for Depression (HRSD-21) between the *n*-3 PUFAs or placebo groups. The points represented the mean HRSD scores and the bars indicated the 95% confidence intervals (CI). The significant differences were observed at week 4 (*p* = 0.004), week 6 (*p* = 0.006), week 8 (*p* = 0.004), and week 12 (*p* = 0.01). The mixed model analysis showed the effect of treatment on HRSD scores may have varied over time and indicated that the interaction between treatment and time may have been marginally significant (F = 2.405, *df* = 5, 74.824, *p* = 0.045).

Overall, a significant decrease in the HRSD score was observed in the *n*-3 PUFAs group compared with the placebo group (Table 1). The evolution trend in the HRSD score for both groups, in each week, is presented in Figure 2. The mixed model analysis showed that the effect of treatment on HRSD scores may have varied over time, according to the interaction between treatment and time, which may have been marginally significant (F = 2.405, *df* = 5, 74.824, *p* = 0.045). Furthermore, the main effect of time was exceptionally significant (F = 7.255, *df* = 5, 74.824, *p* < 0.001), illustrating that HRSD scores changed greatly over time. Additionally, the main effect of treatment was also significant (F = 6.381, *df* = 1, 58.845, *p* = 0.014), showing that HRSD scores differed between treatment groups, particularly starting from week 4. Even though the *n*-3 PUFAs group, compared to the placebo group, had a relatively higher remission rate (26.7% vs. 10%, *p* = 0.095) and response rate (23.3% vs. 6.7%, *p* = 0.145), no significant difference was discovered.

### 3.4. Comparison of the Erythrocyte n-3 PUFAs Levels and the Common Biochemical Parameters

For the erythrocytes *n*-3 PUFAs composition, there were no significant differences observed in arachidonic acid (AA) (*p* = 0.30), EPA (*p* = 0.60), and DHA (*p* = 0.22) levels after 12 weeks of study between the *n*-3 PUFAs group and placebo group. However, comparing the baseline and week 12 within the treatment groups, significant differences were observed in AA, DHA, and EPA levels in both *n*-3 PUFAs group (AA, (pre–post difference: 2.24, *p* < 0.001), DHA, (pre–post difference: 2.18, *p* < 0.001); EPA, (pre–post difference: 1.38, *p* = 0.015)) and placebo group (AA, (pre–post difference: 1.4, *p* = 0.004), DHA, (pre–post difference: 1.0, *p* = 0.018); EPA, (pre–post difference: 0.92, *p* = 0.040)), respectively.

As for the common biochemical parameter values, there was no significant difference in the biochemical parameters that represent the blood lipid profile, kidney function, and liver function between the two groups. However, the biochemical parameter APTT, which represents the blood coagulation level, exhibited a significant difference between these two groups after 12 weeks of intervention (*p* = 0.005). The N-3 group appeared to have a higher APTT level than the control group.

## 4. Discussion

Overall, the key findings of the study show that *n*-3 PUFAs monotherapy for 12 weeks significantly reduced the depressive symptoms of MDD patients. The findings are in line with the recent clinical studies and meta-analysis indicating that *n*-3 PUFAs exhibit a strong positive impact on depressed patients, which can help alleviate their depressive symptoms [8,28,37,47,49,50]. Interestingly, *n*-3 PUFAs did not show an immediate influence on depression status until week 4. This outcome correlates with previous studies [47,51] which point out that the impact of *n*-3 PUFAs might take some time to present. Even though a significant reduction in HRSD scores was observed in this study, no significant difference was detected in the remission rate and the response rate between these two groups, which is consistent with findings from previous studies [42,52]. The negligible changes in remission and response rates between groups may lead to marginal significance of the interaction between treatment and time in this study. Moreover, the main cause of these inconsequential outcomes could be attributable to the limited sample size. A more substantial sample size might be required to effectively illustrate the treatment impact of *n*-3 PUFAs. Another possible explanation could result from the impact of inadequate patient adherence. The poor compliance of the patients complicates data collection and processing. This insufficient adherence is often accompanied by data gaps and potentially distorts the statistical computations of the remission and response rates. Future research may explore the benefit of incorporating strategies to enhance adherence, such as collaborative care models involving pharmacists [53].

For the biochemical parameter analysis, an increase in the *n*-3 PUFAs composition was observed, particularly in the levels of EPA and DHA. The increase in the *n*-3 PUFAs level, driven by *n*-3 PUFAs supplementation, is consistent with previous studies that identified a response biomarker in managing MDD [54]. Despite the comparable erythrocytes’ *n*-3 PUFAs levels between groups, we believe that this observation may be attributed to several factors, which remain potential limitations. The natural fluctuations in erythrocyte PUFA levels observed in both groups may be attributed to individual metabolic differences and lifestyle changes. Additionally, abnormal fatty acid metabolism and individual responses to *n*-3 PUFAs have been implicated in individuals with multi-episode schizophrenia [55] and MDD comorbid with obesity [56]. Even though the disparity between *n*-3 PUFAs and placebos failed to attain clinical significance, this study did not access the potential downstream effects through the production lipid mediator. Recent studies revealed that MDD patients supplemented with *n*-3 PUFAs have demonstrated a greater increase in lipid mediators such as 18-hydroxyeicosapentaenoic acid (18-HEPE); 13-hydroxydocosahexaenoic acid (13-HDHA), which are associated with improved clinical outcomes [57]. Furthermore, another study indicated EPA enhanced plasma eicosapentaenoylethanolamide (EPEA) levels, a PUFAs-derived mediator that is positively related with clinical remission in MDD patients [58].

Moreover, the mechanism behind variations in *n*-3 PUFA uptake into erythrocytes among individuals remains poorly understood. The observed increase in erythrocyte *n*-3 PUFA levels may also be attributed to the release of stored fatty acids from tissues. Metabolic alteration during the 12-week study period could have influenced the release of stored *n*-3 PUFAs into the bloodstream [59,60,61]. Typically, low DHA intake and higher EPA are often associated with reduced arachidonic acid conversion into proinflammatory mediators, whereas the higher DHA (>1.6 g/d) might promote peroxidation [62,63,64]. Despite the standardized dose of DHA (1.1 g/day) given in this study, the individual responses to PUFAs supplementation can vary substantially. Moreover, it has been suggested that high consumption of *n*-6 PUFAs, especially those high in arachidonic acid, may negate the anti-inflammatory effect of *n*-3 PUFAs [65]. Furthermore, although participants were instructed to maintain their usual diets throughout the study, uncontrolled dietary intake of *n*-3 PUFA-rich foods, such as fish or nuts, which are common in daily diets, may have occurred unintentionally [66]. Despite the fact that participants were advised against consuming additional supplements, and that dietary habits were monitored, subtle variations in dietary intake could have occurred. 

N-3 PUFAs were well tolerated by the participants throughout the study, with no major adverse side effects reported. Specifically, *n*-3 PUFAs showed no significant impact on blood lipid profile, kidney and liver function, or any other bodily functions. This outcome correlates with previous clinical studies, which all obtained similar results showing that adverse side effects of *n*-3 PUFAs should not be a concern during the treatment [67,68,69]. However, as for the analysis of APTT level, the *n*-3 group was significantly higher than the control group. This outcome does not correlate with previous studies [70,71]. Observing this outcome might be related to the possible impact of concurrent medication, the complex interaction of *n*-3 PUFAs and other medications on coagulation markers including APTT was not assessed in the study. Despite this, neither the treatment group nor the placebo group’s APTT levels changed significantly before or after the 12-week treatment, indicating that the treatment had no impact on the blood clotting ability. The results thus reinforced the feasibility of using *n*-3 PUFAs as monotherapy for depression.

Our research exhibits several notable strengths. First, the follow-up period of this study is longer than most previous studies. It can provide a more comprehensive view of the impact of *n*-3 PUFAs on depressed patients. Second, we used the ITT approach for the data collection process, which can minimize the possibility of bias by avoiding the effects of crossover and dropout. Third, given that it involves multiple centers, it may contribute to a greater diversity within the study population. On the other hand, this study has some limitations. First, a significant limitation is the small sample size, which encompassed only sixty participants, and thus it was inadequate to comprehensively assess the effectiveness of *n*-3 PUFAs. Second, the gender imbalance should be noted. There are more females than males among the participants; thus, our findings may not apply to the entire population of individuals with MDD. Third, the management of the placebo response warrants consideration of the control of the placebo response. Patients might show a placebo response that may influence the remission and response rate. Despite a relatively longer treatment period of three months compared to previous studies, the lack of a follow-up period is attributed to a potential study constraint. Future studies with a more extended follow-up period are necessary to fully evaluate the long-term effects on remission and recurrence symptoms. Furthermore, this study relied solely on the clinician-rated scales like HRSD for depressive symptoms assessment, which may limit the overall evaluation. Incorporating patients’ rating scales, such as Montgomery–Åsberg Depression Rating Scale (MADRS), Patient Health Questionnaire (PHQ 9) or other patient-reported outcome measures (PROMs) may provide more comprehensive perspectives of treatment effects and reduce potential biases. Fourth, there is a lack of comprehensive patient characteristics including the absence of data on comorbidities such as medical condition and genetic variation. This could potentially affect treatment outcomes.

## 5. Conclusions

In conclusion, *n*-3 PUFAs is a safe intervention that can improve MDD patients’ depressive symptoms with no major adverse effects. Nonetheless, the remission rate and response rate still present insignificant differences between *n*-3 PUFAs and the placebo group. Therefore, using *n*-3 PUFAs as monotherapy to treat depression needs further corroboration.

## Figures and Tables

**Table 1:** Demographics and outcomes in MDD patients.

	*n*-3 PUFAs (n = 30)	Placebo (n = 30)	*p*-Value
Age ^a^ (years)	35.0 (27.0, 48.0)	35.0 (29.0, 47.0)	0.750
Sex (F: M)	25: 5	25: 5	
Education ^b△^, n (%)			0.680
12 years or less	15 (51.7%)	12 (46.2%)	
More than 12 years	14 (48.3%)	14 (53.8%)	
HRSD Score ^c^			
Baseline	19.17 ± 5.14	20.67 ± 6.43	0.320
Week 2	15.10 ± 5.50	18.80 ± 8.29	0.130
Week 4	13.70 ± 5.88	18.93 ± 7.42	0.004
Week 6	13.93 ± 6.31	19.20 ± 7.78	0.006
Week 8	13.63 ± 6.90	19.07 ± 7.27	0.004
Week 12	13.50 ± 7.01	18.53 ± 7.99	0.010
Responders ^d^, n (%)	8 (26.7%)	3 (10.0%)	0.090
Remitters ^d^, n (%)	7 (23.3%)	2 (6.7%)	0.145
Erythrocyte *n*-3 PUFAs ^a,e^			
AA level, %			
Baseline	1.90 (0.93, 2.63)	2.05 (1.22, 3.44)	0.440
Week 12	4.14 (3.04, 4.94) ***	3.81 (2.91, 1.98) **	0.301
EPA level, %			
Baseline	0.67 (0.43, 0.95)	0.45 (0.27, 1.17)	0.574
Week 12	1.46 (0.71, 2.37) *	1.75 (0.58, 3.30) **	0.600
DHA level, %			
Baseline	1.02 (0.59, 2.20)	1.22 (0.56, 1.98)	0.620
Week 12	3.47 (2.42, 3.97) ***	3.07 (1.48, 3.68) *	0.220

Data presented as mean ± SD or median (25th percentile, 75th percentile). The Mann–Whitney U-test ^a^ was used to compare age and erythrocyte *n*-3 PUFAs between groups. The chi-square test ^b^ was used to examine the education differences. An independent *t*-test ^c^ was used to assess HRSD scores variance between groups. Fisher’s exact test analysis ^d^ examined response and remission rate. Wilcoxon’s Signed Rank test ^e^ was used to evaluate the differences before and after treatment in erythrocyte *n*-3 PUFAs. ^△^ Information is missing for some subjects (n = 55). * Indicates statistical significance at *p* < 0.05; ** indicates statistical significance at *p* < 0.01; *** indicates statistical significance at *p* < 0.001. Abbreviations: MDD: major depressive disorder; *n*-3 PUFAs: omega-3 polyunsaturated fatty acids; HRSD: Hamilton Rating Scale for Depression; AA: arachidonic acid; DHA: docosahexaenoic acid; EPA: eicosapentaenoic acid.

## Data Availability

All datasets developed and analyzed for this study are available from the corresponding author upon reasonable request, but they are not publicly accessible for ethical reasons.

## Supplementary Materials

The following supporting information can be downloaded at: https://www.mdpi.com/article/10.3390/nu16213688/s1. File S1: CONSORT Guideline.
